# Integral Phylogenomic Approach over *Ilex* L. Species from Southern South America

**DOI:** 10.3390/life7040047

**Published:** 2017-11-22

**Authors:** Jimena Cascales, Mariana Bracco, Mariana J. Garberoglio, Lidia Poggio, Alexandra M. Gottlieb

**Affiliations:** 1 Departamento de Ecología, Genética y Evolución, Facultad de Ciencias Exactas y Naturales, Universidad de Buenos Aires—IEGEBA (UBA—CONICET), C1428EHA Ciudad Autónoma de Buenos Aires, Argentina; jcascales@ege.fcen.uba.ar (J.C.); marianabracco@unnoba.edu.ar (M.B.); marianajg@ege.fcen.uba.ar (M.J.G.); lidialidgia@yahoo.com.ar (L.P.); 2 Consejo Nacional de Investigaciones Científicas y Técnicas (CONICET), Godoy Cruz 2290, C1425FQB Ciudad Autónoma de Buenos Aires, Argentina

**Keywords:** multilocus phylogenomics, networks and splits graphs, non-coding regions, plastomes, supernetwork, character evolution

## Abstract

The use of molecular markers with inadequate variation levels has resulted in poorly resolved phylogenetic relationships within *Ilex*. Focusing on southern South American and Asian species, we aimed at contributing informative plastid markers. Also, we intended to gain insights into the nature of morphological and physiological characters used to identify species. We obtained the chloroplast genomes of *I.* *paraguariensis* and *I. dumosa*, and combined these with all the congeneric plastomes currently available to accomplish interspecific comparisons and multilocus analyses. We selected seven introns and nine IGSs as variable non-coding markers that were used in phylogenomic analyses. Eight extra IGSs were proposed as candidate markers. Southern South American species formed one lineage, except for *I. paraguariensis*, *I. dumosa* and *I. argentina*, which occupied intermediate positions among sampled taxa; Euroasiatic species formed two lineages. Some concordant relationships were retrieved from nuclear sequence data. We also conducted integral analyses, involving a supernetwork of molecular data, and a simultaneous analysis of quantitative and qualitative morphological and phytochemical characters, together with molecular data. The total evidence tree was used to study the evolution of non-molecular data, evidencing fifteen non-ambiguous synapomorphic character states and consolidating the relationships among southern South American species. More South American representatives should be incorporated to elucidate their origin.

## 1. Introduction

The cosmopolitan plant genus *Ilex* L. (Aquifoliaceae Bartl.) comprises ca. 600 functionally dioecious species of perennial and deciduous trees and shrubs [1,2]. Most species have ornamental value, while several others have medicinal and nutritious properties [3]. Tropical America and subtropical East Asia are documented centres of diversity for the genus [1,2], which has ample representation in the Neotropics (ca. 220–300 species) but is less abundant towards subtropical South America [1,2,4]. Particularly, less than 15 species have been described across eastern Paraguay, southern Brazil and north eastern Argentina, and only two are known for Uruguay [3,4,5,6,7,8]. Among southern South American (sSA) species, *I. paraguariensis* A. St.-Hil.—the “yerba mate” tree—is the most important from a socio-economic perspective. The aerial parts of *I. paraguariensis* are commercialized to prepare a tea-like beverage called “mate”, highly popular in most sSA countries, and lately also in the Middle East. The infusion is appreciated for its distinctive flavour and invigorating and nutraceutical properties, attributed to the high concentration of secondary metabolites [9,10,11]. In the past, *I. argentina* Lillo, *I. brasiliensis* (Spreng) Loes., *I. brevicuspis* Reissek, *I. dumosa* Reissek, *I. integerrima* (Vellozo) Reissek, *I. microdonta* Reissek, *I. pseudobuxus* Reissek, *I. taubertiana* Loes., and *I. theezans* Reissek, were used as adulterants of the “yerba mate” [12]. This led to several phytochemical and morphological studies for species identification [5,6,7,13,14,15,16,17]; nowadays, only *I. dumosa* preparations are promoted as a caffeine-free alternative to the genuine “mate” [18].

The origin and affiliation of sSA *Ilex* species are unclear [19,20,21,22,23,24]. Previous molecular studies showed association with North American, Central American and Asian taxa [20,21,22,23,24]. Still, phylogenetic analyses have been based on few universal nuclear and/or heterologous plastidial markers with dubious resolution power [19,20,21,22,23,24,25,26]. Hence, published phylogenies showed taxon instability, poor backbone resolution and incongruences, leading to unstable relationships [20,21,22,24]. Most phytochemical and morphological studies of sSA taxa were mainly descriptive [3,4,5,6,7,13,14,27], and only a few employed phenetic methodologies [28,29]. However, Loizeau et al. [1] attempted a phylogenetic analysis with morphological characters.

Manen et al. [22] estimated that the age of the most recent common ancestor of the extant *Ilex* species’ plastomes is about 15 million years. Nuclear-based phylogenetic studies in *Ilex* evidenced frequent interlineage and intersectional hybridizations, lineage sorting, gene flow, and inconsistencies with traditional classification [20,21,22,23,24,25,26]. In contrast, plastid-based phylogenetic trees correlated with the geographical distribution of the species without evidencing gene flow, and in some cases, were more consistent with morphologically-based taxonomy [21,22,23,24,25,26]. In this context, plastidial markers emerge as more appropriate tools for studying phylogenetic relationships in this group, and also as a source of valued variable genetic markers. The chloroplast genome has uniparental transmission mode (maternal, in most angiosperms), haploidy, and substantially less gene flow compared to biparentally inherited nuclear genes [30,31,32,33]. The literature abounds in studies focused on detecting variable plastidial markers and in successfully applying them at various taxonomic levels [34,35,36,37,38,39]; however, the early diverging Aquifoliaceae has been scarcely represented [40,41]. Aiming at a forthcoming elucidation of the relationships within *Ilex* through truly informative markers and robust phylogenies, we herein characterized the chloroplastidic genomes of sSA endemic species *I. paraguariensis* and *I. dumosa*, and of the Asian *I. cornuta* Lindl. and Paxt. Then, using all the *Ilex* plastomes currently available, we accomplished interspecific comparisons and multilocus analyses, selecting variable non-coding plastidial markers with which we assessed the phylogenetic relationships among most species from southern South America, together with Euroasiatic representatives. Results were contrasted with those derived from nuclear sequence data analyses. Furthermore, integral analyses were performed including nucleotide sequences, morphological, phytochemical and genotyping data. We implemented two phylogenetic approaches, one that uses traditional dicotomical topologies, and another that does not force tree-like topologies to model relationships (that is, split graphs and supernetworks). The present work allowed the improvement of the existing draft plastome for *I. paraguariensis* [42], but more importantly we contributed a panel of variable markers and we integrated different discrete and quantitative data for the assessment of phylogenetic relationships, acquiring new insights on the evolution of characters used for species identification.

## 2. Materials and Methods 

### 2.1. Plant Material

Leaf samples were provided by the *Ilex* Germplasm Bank at the “Estación Experimental Agropecuaria INTA Cerro Azul” (EEA-INTA-CA; Misiones, Argentina); the Carlos Thays Botanical Garden (JBCTBA; Buenos Aires, Argentina) and the Lucien Hauman Botanical Garden at the “Facultad de Agronomía, Universidad de Buenos Aires” (FAUBA; Buenos Aires, Argentina). For plastome sequencing we used the following accessions of *I. paraguariensis:* EEA-INTA-CA n° 30 (Santa Catarina, Brazil), 217 (Paraná, Brazil) and 224 (Alto Paraná, Paraguay); and tree n° 1837 from the JBCTBA. The sampled accessions of *I. dumosa* were: EEA-INTA-CA n° 222 (Misiones, Argentina), 55 (Paraná, Brazil), 227 (Alto Paraná, Paraguay), and CRV7 (Misiones, Argentina, provided by Dr. PA Sansberro, from IBONE-CONICET). All these samples were chosen to represent plastidic variation detected on a preliminary survey using heterologous microsatellites: two haplotypes in *I. paraguariensis* [43] and four in *I. dumosa* (J Cascales, UBA-FCEN, Argentina, unpub. res.). Detailed lists of species of *Ilex* used throughout this work are presented in Tables S1 and S2. Allopatric taxa (European and Asian) were chosen based on plastid sequence availability from public databases.

### 2.2. Chloroplasts DNA Extraction and Processing

Chloroplasts were isolated from fresh leaves using the Chloroplast Isolation Kit (Sigma-Aldrich, Saint Louis, Missouri, USA) according to manufacturer´s instructions; plastid DNA was extracted using protocols of Diekmann et al. [44] and Shi et al. [45]. Sequence data were obtained for each individual plant using the Whole Genome Shotgun single-end sequencing strategy with the 454 GS FLX+ technology (Roche) at INDEAR (Santa Fe, Argentina). A combination of de novo and reference-guided assembly strategies were performed using Celera Assembler 6.1 [46], Newbler 2.6 and Newbler 2.8 assemblers (Roche). The raw data and the contigs were filtered through BLASTn searches against plastid genomes of *Camellia* L. spp. (NC_020019, NC_022264, NC_022459-63, KF156836 and KF562708), *Coffea arabica* L. (NC_008535) and *Theobroma cacao* L. (NC_014676.2). Mitochondrial and nuclear contaminating sequences were trimmed using Sequencher v4.1.4 (GeneCodes Corp., Ann Arbor, MI 48108, USA). Representative plastomes for each species were generated with consensus sequences across individuals in Sequencher (minimum overlap of 100 bp and minimum match percentage of 90%). Through Sanger sequencing on an ABI 3730xl instrument (Applied Biosystems, Foster City, CA, USA) and using PCR primers designed with Primer3Plus [47] and Primer-BLAST [48] programs as well as primers designed by [49] (Table S3), we verified the junctions between small (SSC) and large (LSC) single-copy regions and inverted repeat regions (IRs), and we filled gaps.

### 2.3. Genomic DNA Extraction and Processing

Total genomic DNA from sSA *Ilex* species (detailed in Table S2) were obtained previously [20]. Fresh leaves of *I. aquifolium* L. and *I. latifolia* Thunb., were processed with the DNeasy Plant Quick Extraction kit (QIAGEN Inc., Duesseldorf, Germany) following manufacturer’s instructions. PCR primers were designed as above or obtained from [50,51] (Table S3), and reaction conditions were as described in Cascales et al. [43]. PCR purifications were done with AccuPrep^®^ Gel purification kit (BIONEER, Daedeok-Gu, Daejeon 306-220, Korea). Sequencing reactions were carried out as above. Genbank accession numbers of the sequences obtained herein and of those downloaded are indicated in Tables S1 and S2.

### 2.4. Plastome Characterization

Genome annotation was performed using CpGAVAS [52] with default parameters. For each protein-coding sequence (CDS), the start and stop codons were established by comparison with the MSWAT alignment [53] and with the annotated sequences of *I. cornuta* (see below). Transfer RNA (tRNA) genes were identified with tRNAscan-SE 1.21 [54] with default settings. All annotations were adjusted using Geneious 7.1.7 (Biomatters, available from http://www.geneious.com). The consensus plastomes were deposited in Genbank (KP016927-KP016928).

A schematic plastome for *I. cornuta* was generated using the 125 sequences publicly available (totalling 105 Kb) by mapping against the complete consensus plastome of *I. paraguariensis,* in Geneious (Supplementary File 1). As no reliable living material of *I. cornuta* is available to us, the gaps were filled with ambiguous bases (N).

Plastome maps were created with OGDraw [55] using standard settings. Global alignments were accomplished with MAFFT multiple aligner 1.3.3 [56] in Geneious, from which identity statistics were derived. Conservation level was assessed via pairwise alignments using mVISTA [57] in LAGAN mode [58] and *Panax schinseng* Nees (=*P. ginseng*, AY582139) as the reference, to make differences more evident.

### 2.5. Non-Coding Marker Selection

A number of *Ilex* species and plastid regions (Table S1) were used to assess the nucleotide variation level of single-locus alignments through the calculation, for each region, of a normalized divergence ratio (=(number of variable sites/number of constant sites)/number of species considered) and the average of the pairwise uncorrected p-distances (p-distance = number of variable sites/total number of sites). The number of variable and constant sites, and p-distances were estimated from single-locus alignments performed in MEGA 6.0 [59]. Those values were used for among-region comparisons visualized with histograms. Previous to the release of plastomes for six Asian *Ilex* species [60], we had selected 7 introns and 10 IGSs as potential phylogenetic markers, using data available at the time (3–14 species for introns and 4–14 species for IGSs; Table S1 and Figure S1). The plastomes generated by Yao et al. [60] were employed for re-testing our marker selection with more species. Thus, the 18 introns were re-scrutinized with 8–20 species, using updated average normalized ratio (=0.0016), and average p-distance (=0.0042) values as references. We also re-evaluated our set of IGSs, and included regions not previously tested. For this extended set of 30 IGSs we used updated thresholds (average normalized ratio = 0.0023, and average p-distance = 0.0067).

### 2.6. Chloroplast Phylogenomic Analyses

We validated the phylogenetic performance of selected markers in the context of other angiosperms. Taxa included were from clades euasterids I (orders Solanales and Gentianales) and euasterids II (orders Aquifoliales, Apiales and Asterales); taxa from Ericales and Malvales were included as outgroups (for accession numbers see Table S1). As selected IGSs proved more variable than introns (average normalized ratios = 0.0049 and 0.0032, respectively), we focused on those regions and we obtained 80 sequences for additional species of *Ilex,* and downloaded other sequences from Genbank (Table S2). PCR amplifications were carried out using conditions described in Cascales et al. [43] and annealing temperatures of 50 or 55 °C; purification and sequencing steps were performed as outlined before. For those species with several sequences per region we generated consensuses in Bioedit [61]. Concatenation and a first round of alignment were performed in Geneious; re-alignments were done in MEGA using MUSCLE [62] with default settings. Best-fitting substitution models were determined in MEGA. Phylogenomic analyses of multilocus matrices were performed under the Maximum Likelihood (ML) criterion with the same program. The ML searches used the BioNJ topology as the initial tree, and the branch swapping filter was set to strong. Bootstrap support values were estimated in MEGA with 500 pseudoreplicates. Also, a multilocus IGS matrix, generated solely for *Ilex,* was analysed through ML as explained above, but without considering a root (i.e., unrooted). As well, this matrix was analysed through the Neighbour-Net algorithm (NN; [63]) in SplitsTrees 4 [64] with the following settings: uncorrected p-distance; edge fitting as ordinary least squares; equal angle as the chosen splits transformation; least squares to modify weights; and four maximum dimensions as the filtering option. Fit and least squares fit (LSfit) values were computed by SplitsTrees, between the pairwise distances in the graph and in the matrix. The split graph generated in the NN yields a visual representation of conflicting signals in the data by presenting them as a series of parallel edges [63].

### 2.7. Nuclear Sequences Analyses

To compare relationships inferred from plastidial data (maternal inheritance) with nuclear information (biparental inheritance), we downloaded from Genbank nuclear rDNA sequences (ITS) from 17 *Ilex* species and sequences of the nuclear encoded protein gluthamine synthetase (nepGS) from 15 species (Table S2). The nuclear information was subjected to unrooted ML and NN analyses, as described above.

### 2.8. Supernetwork

This approach uses as input a collection of topologies defined on fully or partially overlapping subsets of taxa, and outputs a graphic that summarizes multiple phylogenies showing on which parts all partial gene trees agreed and where there existed contradicting signals. These are shown as “incompatibility boxes” whose “dimensionality” reflects the number of conflicting signals [64]. The supernetwork approach was implemented solely for *Ilex* species, using as input unrooted binary topologies, namely, the ML phylograms for IGSs (20 taxa), ITS (17 taxa) and nepGS (15 taxa), and an AFLP Neighbour-joining phylogram for 7 taxa (*I. argentina*, *I. brasiliensis*, *I. brevicuspis*, *I. dumosa*, *I. integerrima*, *I. paraguariensis* and *I. theezans*). The latter phylogram was derived from that presented by Gottlieb et al. [20], but solely considering the most inclusive node for each taxon clade and its corresponding branch length. The supernetwork was obtained in SplitsTrees (with default settings) by importing the topologies in newick format (Supplementary File 2).

### 2.9. Total Evidence and Character Analyses

All the information available was integrated in a simultaneous analysis in order to find the most parsimonious phylogenetic hypotheses [65]. Thus, a combined matrix was constructed for the 10 species from Southern South America, and a dummy outgroup; it involved 21 quantitative and qualitative morphological characters (external and histological) mostly related to anatomical features of female and male flowers, and 12 phytochemical characters, all extracted from published information (Table S4) [4,13,14,15,17,29,66,67,68,69,70], plus 9702 molecular characters from IGS (7727 bp), ITS (689 bp), and nepGS (813 bp) sequences, and 473 AFLP bands (coded as presence/absence) (Supplementary File 3). AFLP data were extracted from A.M.G. personal data matrix. Phylogenetic searches were conducted in TNT [71], using equally weighted parsimony and exact searches; gaps were treated as a fifth state. The range variation of each quantitative character was standardized as equivalent to one step of a discrete character (i.e., a change between the two most dissimilar states in each continuous character has the same cost as one step in a discrete character) [72]. Group support was evaluated with 1000 pseudoreplicates for both bootstrap and jackniffe (*p* = 0.36) resampling in TNT. Results were used to analyse character evolution of morphological and phytochemical features.

## 3. Results

### 3.1. Analyses and Characterization of Plastomes

The NGS sequencing yielded 111 K reads for *I. paraguariensis* and 97 K for *I. dumosa,* with an average length of 618 bp. The complete consensus plastome assembled for *I. paraguariensis* (38 X average coverage) and the draft for *I. dumosa* (8 X), showed the typical quadripartite structure and gene content (Figure S2, Table S5), and similar proportions of structural features (58% of genes and 42% of non-coding sequences; Figure S2). The deduced schematic plastome for *I. cornuta* followed this structure, although showed 16.8% of missing data (Figure S2).

**Figure 1:**
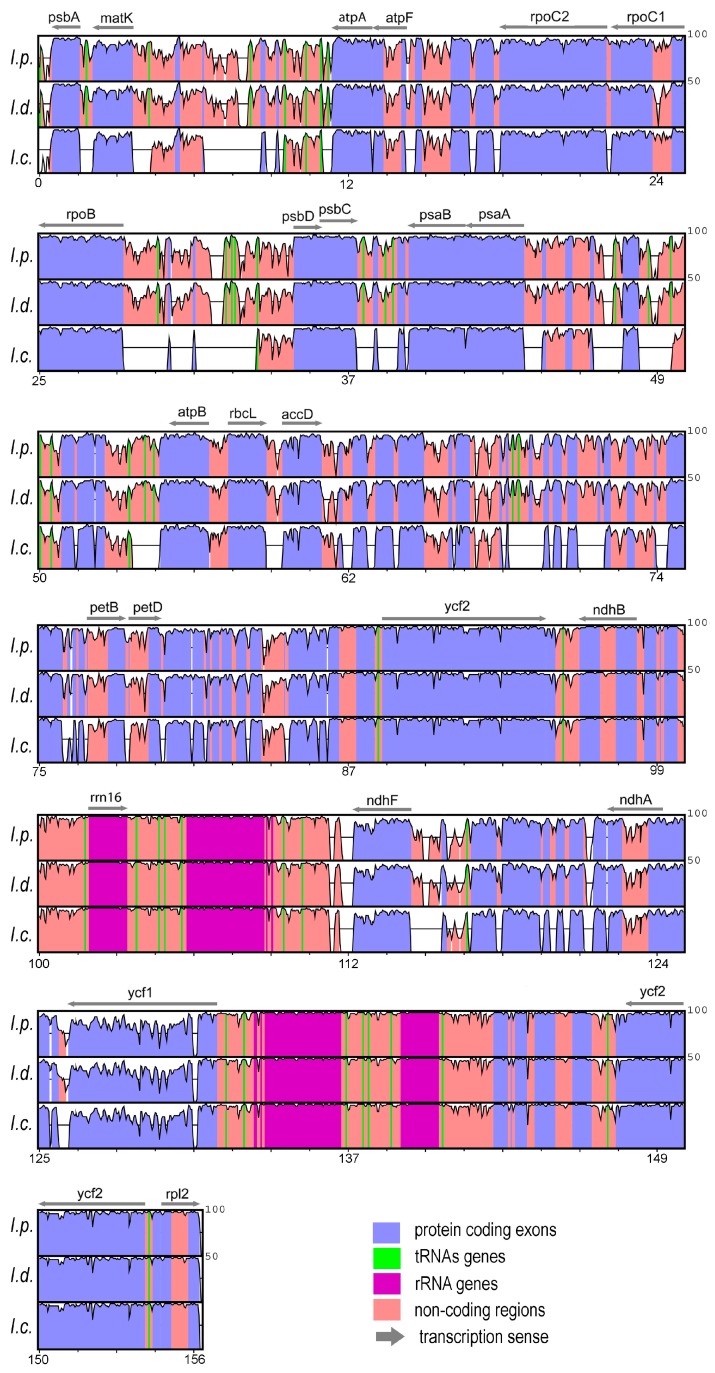
Sequence identity among plastomes of *I. paraguariensis*, *I. dumosa* and *I. cornuta*, using *Panax ginseng* as the reference. For each species (indicated by its initials), the peaks and valleys depict sequence identity estimated against the reference sequence, between 50% (lower line) and 100% (upper line). The horizontal axis shows the position (in Kbp) along the linear chloroplast. Only some features are indicated in each block for guidance; full maps are shown in Figure S2. Regions with missing data are indicated by empty spaces. Grey arrows above each panel denote transcription sense for each gene. Plastomic regions are colour coded as follows: light blue, protein coding (exons); green, tRNAs genes; violet, rRNA genes; pink, non-coding regions (introns and intergenic spacers).

The three plastomes aligned spanned 158.3 Kbp; as expected, the most conserved regions were—in decreasing order—the ribosomal genes, the tRNA genes, the CDS, the introns and IGS regions (Figure 1). Sequence identity between *I. paraguariensis* and *I. dumosa* was 99.01%. The seven Asian species plastomes exhibited 95.50% similarity on average, and when *I. cornuta* was excluded that value raised to 99.10%. The plastome of *I. polyneura* was the most similar to *I. paraguariensis* (98.77%) and *I. dumosa* (98.76%). The global average similarity was 96.2% among the nine species of *Ilex* (rising to 99.0% when *I. cornuta* was excluded).

### 3.2. Marker Selection

**Figure 2:**
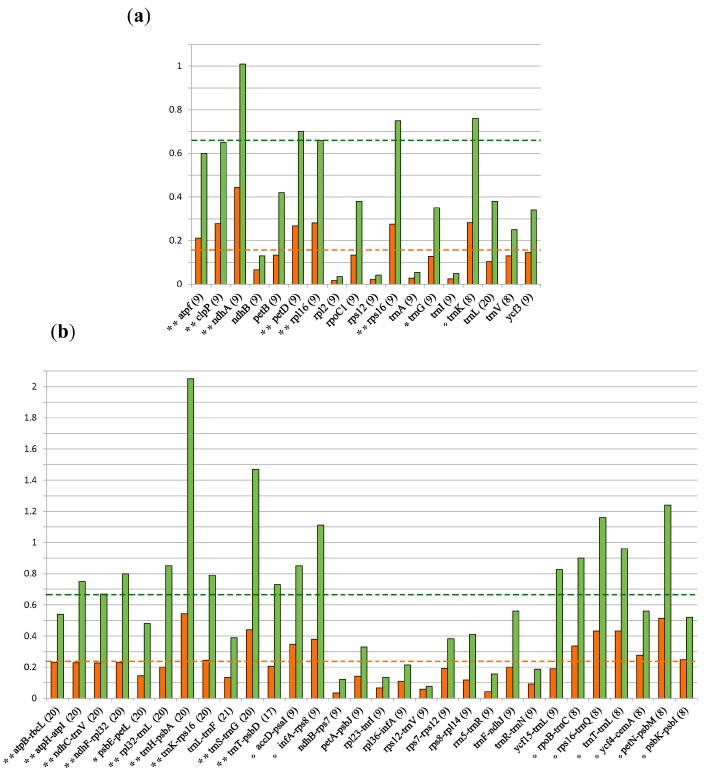
Survey of nucleotide variation of non-coding regions. Histograms showing the normalized ratio expressed as percentage, in orange, and the average uncorrected p-distance, expressed as percentage, in green. The number of *Ilex* species used is indicated between brackets. * Marks a region selected as in Figure S2; ** mark regions validated with current sampling; ° mark regions selected solely under current sampling. (**a**) Values estimated from 18 single-loci intron alignments; (**b**) Values estimated from 30 single-loci IGS alignments.

The addition of data from Yao et al. [60] modified our original selections. With a minimum of eight species surveyed per region, the most variable introns were those for genes *atpF*, *clpP*, *ndhA*, *petD*, *rpl16*, *rps16* and *trnK* (Figure 2a), the latter replaced intron *trnG*. Our original IGS survey indicated that *atpB-rbcL*, *atpH-atpI*, *ndhC-trnV*, *ndhF-rpl32*, *psbE-petL*, *rpl32-trnL*, *trnH-psbA*, *trnK-rps16*, *trnS-trnG* and *trnT-psbD* qualified as variable markers for *Ilex* (Figure S1). With the inclusion of the plastomic data of Yao et al. [60] most markers were validated, but *psbE-petL*. Among the newly tested regions, eight qualified as additional variable markers (namely, *accD-psaI*, *infA-rps8*, *rpoB-trnC*, *rps16-trnQ*, *trnT-trnL*, *petN-psbM*, *ycf4-cemA* and *psbK-psbI*; Figure 2b).

The inspection of single-locus alignments from validated markers detected several small structural rearrangements. For instance, *trnS-trnG* exhibited a 44 bp-long palindromic region, and 15 bp-long non-tandem duplications shared by sSA *I. integerrima*, *I. brasiliensis*, *I. microdonta*, *I. pseudobuxus*, *I. brevicuspis* and *I. theezans*, and by Asian *I. wilsonii* and *I. szechwanensis.* Within *rpl32-trnL*, a 101 bp deletion was solely detected for *I. paraguariensis*; the deleted region retrieved BLAST hits exclusively with *Ilex* plastomes. As well, a small inversion (44 bp) was shared by *I. integerrima*, *I. brasiliensis*, *I. brevicuspis* and *I. theezans*.

### 3.3. Phylogenomic Analyses

The phylogram inferred from the selected intronic regions showed that the clades euasterids I and II were clearly defined (BSV ≥ 98%; Figure S3a). *Ilex paraguariensis* and *I. dumosa* (BSV = 80%) appeared as sister group (BSV = 89%) of the Asian clade formed by *I. cornuta, I. latifolia* and *I. delavayi.* The other four Asian species branched as sister group (BSV = 81%). The phylogram derived from the 9 IGS also recovered the clades euasterids I and II (BSV = 100 and 87%, respectively; Figure S3b). Southern South American species *I. brevicuspis* and *I. theezans* (BSV = 97%) plus *I. brasiliensis*, *I. taubertiana*, *I. pseudobuxus*, *I. integerrima* and *I. microdonta* appeared robustly related (BSV= 99%). Whereas, *I. argentina* and *I. dumosa* received moderate support (BSV = 70%) as sister to the former; *I. paraguariensis* was located as sister taxa to all sSA (BSV = 93%). Two other clades were formed, the one related to sSA species (BSV = 92%) involved Euroasiatic taxa *I. kaushue*, *I. pentagona*, *I. latifolia*, *I. cornuta*, *I. aquifolium* and *I. delavayi* (BSV = 99%), and the other comprised *I. polyneura, I. pubescens, I. wilsonii* and *I. szchewanensis* (BSV = 98%).

**Figure 3:**
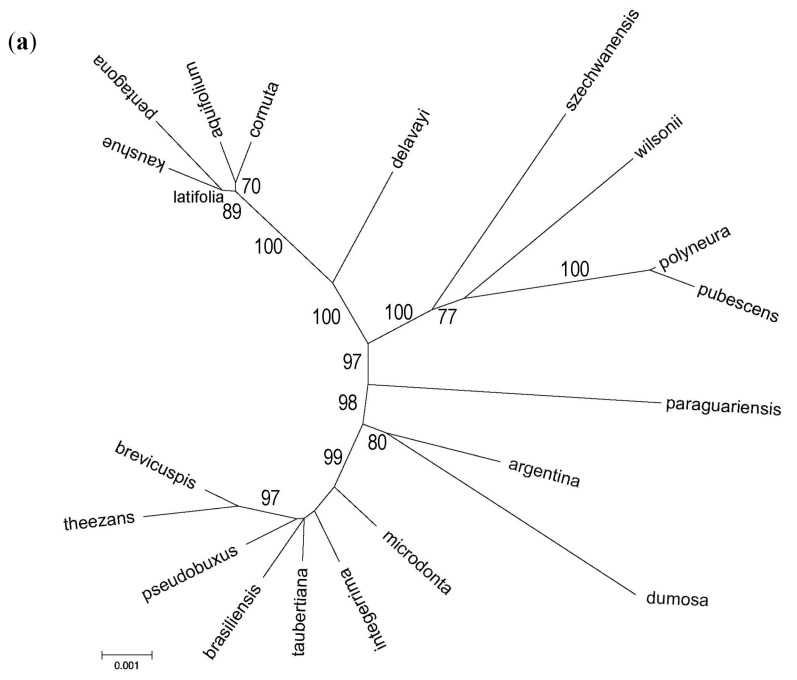

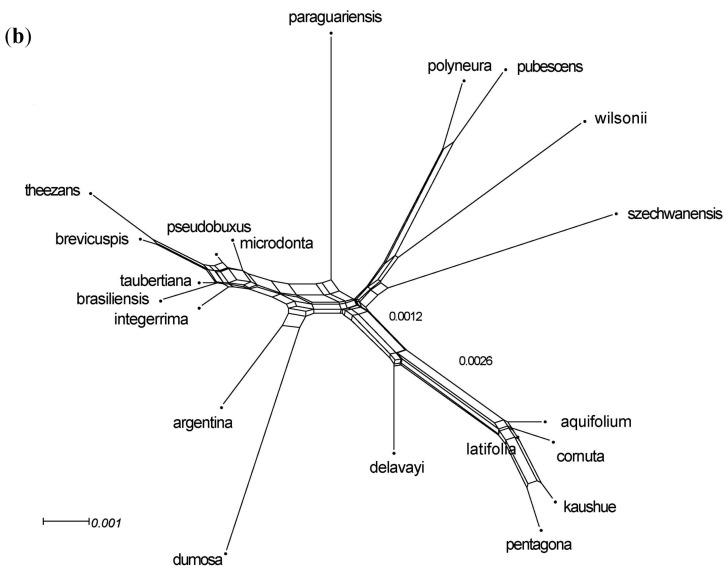
Genetic relationships among 20 *Ilex* species computed from the multilocus IGS dataset. The species were named using the specific epithet alone, for simplicity. (**a**) The Maximum Likelihood unrooted phylogram (lnL = −13,104.12) was inferred applying a GTR model [73], and a discrete Gamma distribution was used—with five categories—to model evolutionary rate differences among sites (G = 0.092); all positions with <50% coverage were eliminated, thus 7655 bp were considered. Bootstrap values >50% (500 pseudoreplicates) are shown. Branch lengths are in number of substitutions per site; (**b**) Neighbour-Net split graph based on uncorrected p-distance. All positions were used to derive the network (7862 bp; fit = 97.36; LSfit = 99.89). The scale bar is in genetic distance units. The numbers indicated the edge weight, which is proportional to the edge length.

The multilocus IGS alignment solely for *Ilex* species spanned 7862 bp, having 6.27% of missing data. Average genetic distance between sSA and Euroasiatic species (uncorrected p-distance = 0.0103) was 1.8 to 1.2 times higher than that calculated for each group, respectively. Though consistent with the rooted ML topology (Figure S3b), the unrooted ML clearly showed that *I. argentina*, *I. dumosa*, *I. paraguariensis*, *I. delavayi*, *I. wilsonii* and *I. szchewanensis* had the largest branches and intermediate positions (Figure 3a). The NN network agreed in that, and also highlighted conflicting signals among sSA taxa and among *I. aquifolium*, *I. cornuta*, *I. kaushue*, *I. pentagona* and *I. latifolia* (Figure 3b).

### 3.4. Nuclear Sequence Analyses

The unrooted ML phylogram and the NN derived from ITS matrix showed that *I. paraguariensis* and *I. dumosa* (BSV = 98%) occupied an intermediate position, strongly supported by the data (shown by the heaviest edges), and exhibited the largest branches (Figure S4a,c). NepGS gene network failed to discriminate the species according to their geographic origin. The unrooted ML phylogram and the NN yielded three robust groups (BSV ≥ 94%): *I. dumosa* and *I. argentina*, *I. pseudobuxus* and *I. microdonta*, and *I. integerrima*, *I. brasiliensis*, *I. brevicuspis* and *I. theezans*. Remaining nodes were unsupported (Figure S4b,d).

### 3.5. Integrated Data

**Figure 4:**
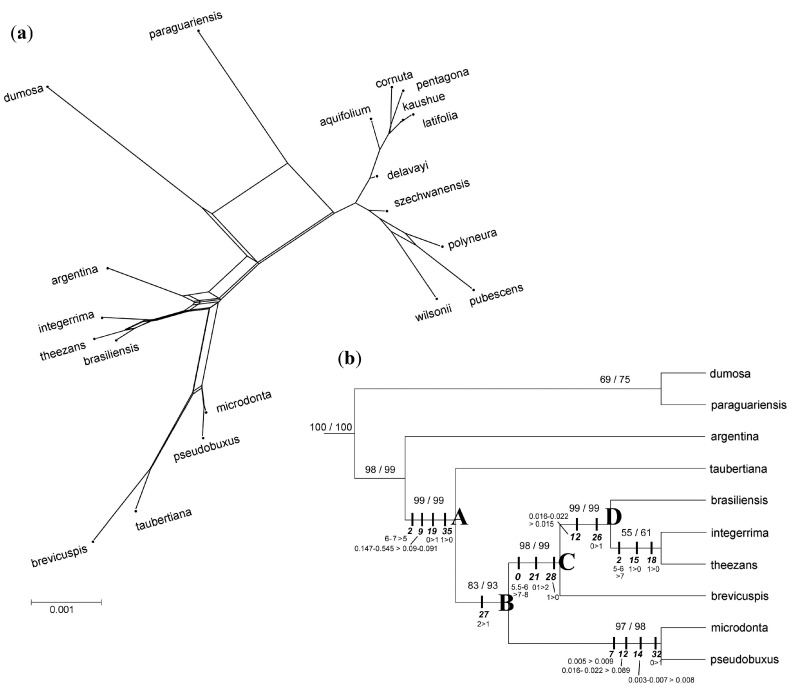
Integral phylogenetic analyses for *Ilex* species. The species were named using the specific epithet alone, for simplicity. (**a**) The supernetwork topology. The scale bar represents edges weights; (**b**) The total evidence most parsimonious topology for sSA species (score = 1689.06; CI = 0.736; RI = 0.471). Numbers above branches are boostrap/jackkniffe support values; non-ambiguous synapomorphic morphological and phytochemical character state changes (not standardized) are indicated at each internal node.

The supernetwork generated from molecular data showed an almost tree-like structure for Euroasiatic species (Figure 4a), but showed conflicting signals, particularly in the relationship among *I. argentina*, *I. dumosa* and *I. paraguariensis*, and the placement of *I. brevicuspis*.

Total evidence approach was implemented solely for sSA species, rendering a single most parsimonious tree (Figure 4b) where most nodes received good support (BSV/JK ≥ 83/93%), except the relationship of *I. paraguariensis* and *I. dumo*s*a* which was moderately supported (BSV/JK ≥ 69/75%), and that of *I. integerrima* and *I. theezans* which received poor support (BSV/JK = 55/61%). When morphological and phytochemical characters were optimized over the tree, 15 non-ambiguous synapomorphies were evidenced. For the members of clade A, the 1.2–1.7 times reduction in fruit size (char. 2), the absence of colleters (external secretory structures involved in the protection of buds and young leaves) on leaf laminae (char. 35), and the presence in female flowers of papillae over the epidermis of staminoidia (arrested stamens shown by female flowers; char. 19), are states that had been inherited from the common ancestor although *I. microdonta* had secondarily lost papillae, and *I. integerrima* exhibited lachrymiform colleters (homoplasies). There also had been a 2–6 times decrease in the content of a caffeoyl derivative (3.5 dicaffeoylquinic acid; char. 9) before the divergence of the seven species. The hypothetical ancestor of clade B may have produced dorsally striated pyrenes (char. 27), instead, the smooth pyrenes shown by *I. integerrima*, *I. brevicuspis* and *I. pseudobuxus* may had been acquired in parallel. Descendants of clade C, shared an increment in their leaves length (1.3 times; char. 0) and in the innervation of the gynoecium (either functional or aborted; char. 21), and exhibited rostrated pistilloidia (aborted pistils generated by male flowers; char. 28). The obtuse leaf apex (char. 26) and the decrease in the content of the flavonoid rutine (up to 1.5 times; char. 12) shared by members of clade D represented novel homologies. Similarly, the increase in fruit size (1.2–1.4 times; char. 2) and the absence of papillae from the epidermis of petals (char. 15) and stamens (char. 18) shown by *I. theezans* and *I. integerrima* may represent homologous changes for the two species, but convergent features for *I. dumosa* and *I. argentina*. Since divergence from the common ancestor (node B), the hypothetical ancestor of *I. microdonta* and *I. pseudobuxus* had experienced an increment in the content of the flavonoids rutine (6 times; char. 12) and kampferol (1.1–2.7 times; char. 14), and in the content of caffeic acid (1.8–2.3 times; char. 7). Also, the low concentration of methylxantines (caffeine; char. 32) was a homologous feature for these two species.

## 4. Discussion

We herein contributed the first complete plastomes for sSA *Ilex* species, a draft for the ornamental Asian species *I. cornuta*, and plastidic information from several other congeneric species. These data complement that for species from China [60]. In concordance with the conserved nature of the chloroplasts [35,40,74], the plastid genomes of *Ilex* species displayed synteny even with the distantly related *P. ginseng* (Apiales). This agrees with the high overall conservation visualized (Figure 1), the high interspecific sequence identity (average = 96.2%) and the relatively short branches shown by the Aquifoliaceae in the phylogenomic analyses (Figure S3a,b). Moreover, we found that *Ilex* plastomes exhibited the ancestral gene order seen among unrearranged angiosperms like *Nicotiana* (Solanaceae) [36,75,76]. We detected that the draft plastome depicted for *I. paraguariensis* by Debat et al. [42] showed the LSC region with the two large overlapping inversions (comprising a 22.8 Kb region and a 3.3 Kb nested inversion) described for *Lactuca* (Asteraceae) by Timme et al. [77]. Thus, caution should be taken not to confound those features as relevant to *Ilex*. Large and small-scale duplications, inversions, and indel events, and pseudogenization are known to have diversified angiosperm plastomes [39,74,76]. Herein, only small structural rearrangements (<200 bp) were detected, such as an autapomorphic deletion within *rpl32-trnL* for *I. paraguariensis,* and a synapomorphic deletion within *trnK-rps16* for *I. polyneura*, *I. pubescens* and *I. wilsonii*. Also, convergent duplications were found, for instance, within *trnS-trnG*. We anticipate that as more species are surveyed, more rearrangements will be unveiled, so that their phylogenetic information could be appropriately extracted.

We are aware that the coverage obtained herein is lower than expected by NGS, and in comparison with similar studies [60,78,79]. Even though identical experimental procedures and bioinformatic pipelines were used for both species, a low proportion of chloroplastidic reads were obtained (<7%). This could be accounted for by unnoticed experimental artefacts combined with the abundance of cytosolic metabolites that may interfere with intact chloroplast isolation and/or with downstream procedures. A severe interference from cytosolic metabolites was appreciated during nuclear staining for cytoflow measurements, not only in these species [80] but also in *Coffea* [81]. Besides, contamination with nuclear and/or mitochondrial DNA cannot be discarded, as was also found in other species [82]. In spite of these negative effects, the data gathered and curated here enabled the generation of coherent plastomic maps.

As mentioned before, the use of universal plastidial markers for *Ilex* phylogenetics yielded poorly resolved relationships [1,19,21,22,23,24,25,26]. To overcome this caveat, we carefully examined all the plastomic data available for *Ilex* to identify informative markers, which were validated by their ability to resolve well-known clades. Thus, we defined a subset of seven introns that may deserve further investigation, and we detected that the *trnL* intron, which is routinely used in plants [83], resulted inadequate for *Ilex* as it did not add significant information (Figure 2 and Figure S1). Also, we herein selected a panel of nine IGS markers that proved suitable for phylogenetic analyses within *Ilex*. The region *trnH-psbA* resulted the best ranked, although when used as single locus it yielded rather unresolved topologies [24]. Our results corroborate those of Shaw et al. [39,50] in that *ndhC-trnV*, *ndhF-rpl32*, *rpl32-trnL*, and *trnT-psbD* are among the most variable regions. Surprisingly, the widely used *trnL-trnF* had a poor performance in face of the evidence provided. Other eight IGS are proposed here as alternative markers.

The intronic and IGS phylogenomic analyses recovered concordant hypotheses in which the Aquifoliales (*Ilex* + *Helwingia*) appeared as the sister group of the Apiales and Asterales. A similar result was obtained by Yao et al. [41], although the placement of the Aquifoliales was misinterpreted there as “basal”. The marker regions proposed by Yao et al. [60], most of which consisted of large coding and non-coding regions, were analysed here and yielded an average normalized variation ratio (0.0025; Figure S5) that was lower than the obtained from our panel of selected introns (0.0029) and IGS (0.0032). Still, we agreed in considering the intron of *ndhA* as a powerful marker (Figure 2). Thus, results presented favoured the use of the set of markers reported here, instead of large compound regions, at least for species level phylogenies.

By the simultaneous analyses of multiple non-coding loci we provided robust evidence for the existence of at least three distinct maternal lineages in southern South America. Wild species *I. brevicuspis*, *I. brasiliensis*, *I. integerrima*, *I. microdonta*, *I. pseudobuxus*, *I. taubertiana* and *I. theezans*, may represent one such maternal lineage. Our results agreed with Manen et al. [22] in that *I. argentina, I. dumosa* and the semi-domesticated *I. paraguariensis* set apart from other sSA species. Moreover, we showed supported intermediate phylogenetic placements and long branches for these species (Figure 4a) that also distinguished them from other sympatric species. This divergence was partially evidenced by metabolomic fingerprints which separated the species into four groups: (a) *I. paraguariensis*; (b) *I. dumosa*; (c) *I. integerrima*, *I. pseudobuxus*, *I. theezans* and probably *I. brasiliensis*, and (d) *I. argentina*, *I. brevicuspis*, *I. microdonta* and *I. taubertiana* [28,84]. *Ilex argentina* is the sole Andean and polyploid species (2n = 80) so far detected in the region [85], as such, it may be reproductively isolated from the rest, configuring another lineage. Whether *I. paraguariensis* and *I. dumosa* represent a single maternal lineage is not definite. We are aware that any phylogenetic inference is conditional on the taxonomic sampling, and that our sampling of *Ilex* is rather restrictive; the inclusion of species, particularly from north-western South America, could help in elucidating the origin and dispersal of *Ilex* in the southern part of the continent, and the genetic basis of the divergence among wild and semi-domesticated species. Still, we herein contribute to a deeper knowledge of the relationships of most sSA species.

The Euroasiatic species *I. aquifolium*, *I. cornuta*, *I. kaushue*, *I. latifolia* and *I. pentagona* represent a well-defined divergent maternal lineage, as judged by the large branches and/or edges shown by this group (Figure 4a). The relationship of *I. aquifolium* with Asian taxa [22] was herein corroborated; this species is native to south, west and central Europe and the Mediterranean [33] and has some medicinal properties reported [86]. *Ilex cornuta*, *I. latifolia*, *I. kaushue* and *I. pentagona* have nutraceutical properties similar to those of “yerba mate”, but based on different secondary metabolites [27,87,88]. Hybrids between *I. cornuta* and *I. latifolia* have been recently reported in Central China [26], evidencing that genetic barriers were not fully developed and, ultimately, questioning the species recognition criteria, as hybridization and introgressions processes are not uncommon within *Ilex* [21,22,25,26]. Noteworthy, no hybridization reports are known for sSA species, so far. In the present study, *I. polyneura*, *I. pubescens*, *I. wilsonii* and *I. szchewanensis* represent another, more divergent, Asian maternal lineage.

Previous phylogenetic analyses of morphological features obtained an unsupported and unresolved consensus topology from which no conclusions could be derived [1]. By means of the total evidence approach we obtained strongly supported and well resolved relationships for most sSA taxa; we also elucidated the nature of several morphological and phytochemical features employed to characterize these species [3,4,5,6,7,13,14,15]. We detected several synapomorphies (i.e., true homologies) and character states that do represent homoplasies throughout the phylogeny. For instance, the papiraceous consistency of leaves and the presence of druses in histological preparations, may obscure relationships. Then, no clear tendency was detected for the length and width (i.e., shape) of the leaves, as both parallel increases and decreases were apparent. Likewise, the abaxial pubescence of leaves resulted in uncertainty for determining groups. Gonzalez and Tarragó [29] distinguished three types of colleters and their locations, but only those present on margins of young leaves retain phylogenetic information. Still, lachrymiform colleters may have arisen by parallel evolution, as they are found in “yerba mate” and *I. integerrima* leaves. This knowledge is valuable in order to generate natural classifications. Although speculative, it seems likely that the peculiar metabolic profile of *I. paraguariensis* may be autapomorphic by means of increased methylxanthines syntheses. The ability to synthesize the glycoside arbutin may have been acquired by sSA species after the divergence of the *I. paraguariensis*-*I. dumosa* lineage, verifying the proposal of Choi et al. [28] for its usage as a biomarker. Further work is needed to unveil the nature of other morphological characters in order to discern truly informative characters from misleading ones. 

## 5. Conclusions

Our study showed that plastid markers usually cited in the literature, are not always the best choice for phylogenetics of a particular group. Whenever possible, a careful survey should be performed to detect regions with the adequate variation level. Until the costs of sequencing complete plastomes become massively affordable, sequencing of non-coding markers with proven variation may be preferable. Moreover, the markers proposed in this study constitute a more effective option, in terms of cost-benefit, than the compound markers suggested by other authors, at least for assessing species level phylogenetics.

As introgression and hybridization processes are documented in *Ilex* species, we herein not only inferred classical tree-like topologies, but also assayed methodological approaches that do not force dicotomical trees to model relationships. These analyses employed several data sources (multilocus plastid and nuclear regions, as well as morphological and physiological characters), some of which were never tested in a phylogenetic framework. In addition, our analyses differ from previous phylogenetic analyses in the improvement of group support values [1,22,24] and highlight the importance of the use of markers with suitable variation levels, and of the combination of different data sources.

Traditionally, species delimitation within *Ilex* was based on morphological characters whose primary homology statements have not been evaluated on phylogenetic trees. More efforts are needed to increase our knowledge on the evolution of such features, and concomitantly, in clarifying the controversial taxonomic system of the genus. As the number of taxa involved rises, we anticipate that more accurate and robust inferences will emerge from the synergic analysis of our set of molecular markers with other sources of data, either molecular or morpho-physiological. Besides, we expect that the contributed knowledge will facilitate the implementation of a chloroplast transformation technology [76,89,90], particularly in those species with nutraceutical attributes. This, in turn, would accelerate the achievement of modern varieties that may fulfil producers and market requirements.

## Supplementary Materials

The following are available online at www.mdpi.com/2075-1729/7/4/47/s1, Figure S1: Nucleotide variation analysis of non-coding regions, Figure S2: Schematic plastomic maps for *I. paraguariensis*, *I. dumosa* and *I. cornuta*, Figure S3: Maximum Likelihood phylogenomic analyses of multilocus data sets performed in the context of other angiosperm taxa, Figure S4: Genetic relationships among *Ilex* species computed from ITS and nepGS nuclear sequences, Figure S5: Nucleotide variation analysis of the plastid regions proposed in Yao et al. [60], Table S1: Genbank accession numbers for intronic and IGS regions for additional species used to evaluate their nucleotide variation level through single-locus alignments, and the phylogenetic performance of the selected markers, in the context of other angiosperms, Table S2: Accession number and geographic origin of each additional *Ilex* species and Genbank accession numbers of IGS, ITS and nepGS regions used, Table S3: Primer pairs used for gap closure, junction verification and intergenic spacer (IGS) amplification, Table S4: Morphological and phytochemical characters considered in the total evidence and character analyses, Table S5: Gene content of the chloroplast genomes of *I. paraguariensis*, *I. dumosa* and *I. cornuta*, Supplementary File 1: Draft plastome for *Ilex cornuta*, Supplementary File 2: Input unrooted trees (networks) in newick format used for supernetwork construction: ML phylograms derived from IGS, ITS and nepGS, and NJ phylogram derived from AFLP, Supplementary File 3: Combined data matrix in TNT format used for total evidence analysis. The data matrices and resulting trees from the ML (Figure 3a) and total evidence (Figure 4b) analyses were uploaded to TreeBASE, at http://purl.org/phylo/treebase/phylows/study/TB2:S21886.
